# The Field of Operative Dentistry

**Published:** 1887-02

**Authors:** H. R. Morton


### The Field of Operative Dentistry.

BY H. R. MORTON.

(Read before the California State Dental Association.)

*Mr. President and Gentlemen:*

I believe it to be the uniform fact, that, in everything wherein man has progressed, he has done so, not by a continuous, but by an intermittent process. If we look at civilization as a whole, we find that it has not advanced continuously from the days of Adam to the nineteenth century; that, on the contrary, there have been times of progress, alternating with times of rest, and even of retrogression. If, instead of civilization as a whole, we view human knowledge, whether scientific or practical, the same fact is conspicuous, and equally plain is it when, instead of knowledge as a whole, we examine its different departments. It seems to me that these periods of rest are necessary to the consolidation of the advance attained, and to the forming of a sound basis for further progress.

In operative dentistry we appear to have entered on one of these periods of rest. Until recently, every meeting, for many years, received accounts of startling novelties—cohesive gold in many varied forms; mallets, hand, spring, and electric; engines with burrs, drills, disks, polishing points, and pluggers; rubber dam, with clamps, forceps, and other appliances; improved plastics, both metallic and non-metallic, together with suggestions innumerable concerning modes of use and indications for the use of these various new resources. Now, though improvement has by no means ceased, it is comparatively inactive. Were your committee on operative dentistry to make a report on the material advance of the past year, we would simply call attention to one or two new matrices and separators.

Instead, however, of discouragement at being obliged to note this relative stagnation of material progress, your committee (one member of it, at least) must express the most marked encouragement. It seems a hopeful sign. There is going on now, throughout the dental world—as may be seen by papers in our journals, and discussions in our Societies—not a material advancement, but a mental advancement, which will, in due time, result in an operative dentistry higher and in every way better than we can now conceive.

Throughout the world, dentists are seeking first principles—building, not high, but deep; not imposing superstructures, but solid foundations. And these foundations, when completed, will bear superstructures grander than anything we have yet seen.

Instead of the cohesive quality of gold, our leading men are discussing the etiology of caries; instead of No. 90 foil, they are talking of the principles on which arrest of decay depends; instead of new forms of rubber dam, they are disputing on the origin and development of the dental follicle; instead of the back-action mallet, they speak of distant reflex symptoms of exostosis and pericementitis.

Modern discussion, then, seeks not the *how*, but the *why*. It aims at the root of the matter. And, as it concerns operative dentistry, it seeks:

First. To define its field of action.

Second. To establish its action within that field on a basis of principle or *reason*.

It being manifestly impossible for your committee, within any decent limits of space and time, to go over the whole of this broad ground, even in the briefest synopsis; I propose to limit myself to a glance of the former branch of the subject—the field of operative dentistry.

Conventionally, we divide dentistry into two branches, operative and mechanical. The operative dentist, like the surgeon, must be a physiologist and mechanic; the mechanical dentist, like the sculptor, must be a mechanic and artist.

The operative dentist—or, as we call him for brevity's sake, the "operator"—deals directly with dental disease. The mechanical dentist or "mechanic" atones, as far as man can atone, for the loss of natural organs, by artificial substitutes.

Thus far there is no dispute. But the moment we attempt to define our definition, we meet with opposition and discussion. If we say that the field of operative dentistry is co-extensive with the field of dental disease, every one admits the fact. But when we attempt to mark off clearly the field of dental disease, we find that no two persons exactly agree on its boundaries.

Here is a woman losing her teeth through an ill-advised pregnancy; here is—what is almost as common—a young man wrecking his incisors and molars by sexual excesses; here is a family of children developing fragile dentures through the operation of that system of hot-house culture which is so fashionable now-a-days. In cases like these, where is the boundary—the line at which dental disease ends, and general derangement begins?

Or take the reverse class of cases. Here is an emaciated woman, whose anæmia, hysteria, and frequently recurring attacks of neuralgia, are mere symptoms of dental exostosis. Here is an irritated, a nodulated, an exposed, or a putrescent pulp, which, giving no local pain, expresses its call for relief through eye, ear, throat, or nose. Here indigestion, chronic diarrhœa, vertigo, and mania, are merely the words of the dens sapientiæ, expressing their inability to erupt. The general practitioner, lost in this labyrinth to which he lacks the key, is powerless—wanders on in darkness, mistaking the symptoms for the disease. Yet here, as before, who shall say where is the boundary—the line which divides the general from the special, the field of medicine from the field of dentistry?

The line indeed is difficult to trace; for the field of the one profession shades imperceptibly into that of the other. It does not follow, however, that they are identical. Day shades insensibly into night; but he who on that account would maintain that day and night are the same, would be liable to combat doubt concerning his sanity.

If, like day and night, physicians and dentists would meet on the debatable ground, great advantage would result to both professions. But this, up to the present time, there has been little disposition to do. Not that dentists have been at any time unwilling to bring their special knowledge and skill to the aid of the general practitioner; but there has been, on the part of the general practitioner, an ignorance of dental matters, whose density only those who have come into contact with it can appreciate; to which certain specimens of the general practitioner have superadded what has been aptly called "the pride of ignorance"—a feeling akin to that with which the Pharisee regarded the Publican—"Stand by thyself; I am holier than thou."

This feeling on the part of what may be called the "lower classes" of the medical profession has come to the front with peculiar boldness during the last two years. Articles have appeared in certain medical journals, heralding to their profession, as great discoveries, commonplaces of which a dentist's office boy would be ashamed to be ignorant; and the announcements of these great discoveries (destined, perhaps, in the estimation of the great discoverers to hand their names down to posterity, alongside of those of Lister, Sims, Virchow, and Pasteur), have been accompanied by explicit statements that the dentists are a set of "mere mechanics," and by dark hints that they should not be allowed to do anything except as directed and supervised by the "superior" practitioners who have made the great discoveries aforesaid. To which I am sorry to add that the dentists, instead of treating these articles and their authors according to their merits—with a smile, a curl of the lip, and a contemptuous silence—have, in one case at least, replied; and, with a humility sometimes read of but seldom seen, they have replied respectfully.

For myself, I have borne these attacks on my chosen profession with the amused complacency commonly shown by one who from a second-story window contemplates the impotent rage of a mongrel cur below.

It has happened that these denunciations of us "mere mechanics, and indifferent ones at that" (of whom I am one of the least advanced and most obscure), has reached me just when I had succeeded in effecting cures to which the medical man is inadequate; the latest effusion—an announcement of the value which creasote and carbolic acid would have as dental pain obtunders, if we ignorant wretches could only somehow be informed of the existence of these substances––coming just as I had discharged, cured, a case of chronic pharyngitis (symptomatic of periodontitis and mal-occlusion) which had resisted the efforts of several medical men.

My sole remark concerning the great discoverers who have called attention to the hopeless ignorance and degradation of our profession, has therefore been: "We see with what class of dentists they associate —*A man is known by the company he keeps.*"

I apprehend that, in spite of the raging of the "mere druggists," who are engaged in general practice, it will be several days—possibly even weeks—before the arrival of the happy time when it will be universally recognized that "the physician should be consulted for an aching tooth as much as for any other aching member; and that, when he has cured it, he should send it to the repair shop of the dentist, if necessary." Nor is this belief at all weakened by recollection of the "few cures" of aching teeth which I have seen attempted by the general practitioner.

In fact, the field of operative dentistry, instead of contracting is daily widening. There was a time—and it was not long ago–– when dentistry was almost wholly prosthetic; when operative dentistry consisted mainly in removing the natural teeth, to make room for what were called, with grim prophetic sarcasm, the patient's "tombstones." But that time is past––never to return.

Whatever portion of the debatable ground may be occupied by the operative dentistry of the future, it will certainly be a liberal one. The dentist has learned—and he will never forget––that it is a part of his duty to trace cause and effect throughout the human system. He may not treat—probably will have no desire to treat—the systematic derangements whence dental diseases spring. Nor may he treat––or even desire to treat—the manifold disturbances which spring from dental disease. All these matters he will undoubtedly leave, in the future as in the past, to the general practitioner or the particular specialist, in whose department the more prominent symptoms show themselves. He will confine his treatment to what may be rigidly called dental disease; this he will combat by every means at his command, both therapeutical and mechanical; but he will not lose sight of those phenomena which are connected with dental disease, both as cause and as consequence; and as far as necessity compels him, he will seek to prevent the disease by removing its causes, and will seek to remove the consequences by curing the disease.

So much for the dentist as a being who "looks before and after," in relation to the diseases which he treats. We now turn to view his field in relation to the organs which are the immediate objects of his care.

Of late, there has been increasingly manifested a disposition to break over the boundaries which have limited the dentist's field in this respect. During the past year, one prominent man has gone so far as to declare that he seeks, not the preservation of the patient's teeth as such, but merely as organs of health and beauty. And this is undoubtedly the stand which will be taken by the dentist of the future. The mania for tooth-preservation at all hazards and under all circumstances has nearly run its course, and in a few years will be confined to the laggards who are always behind the age.

Operative dentistry considers, then, the patient's appearance and health. And the present tendency is, without neglecting the former, to give increased attention to the latter.

Physiologically, the field of operative dentistry is the field of digestion. The dentist seeks to preserve the human teeth, not simply because they are teeth, but because they are organs of digestion. In the last resort, he seeks, not the welfare of the teeth, but the welfare of the patient. And the teeth he saves or destroys, according as their preservation or removal, will most contribute to the patient's well-being.

I anticipate, in the near future a discussion of the subject of digestion, oral, gastric, and intestinal, and of the inter-relations of these phases of the digestive act, more thoroughly and more satisfactorily than we have yet heard. Already the saliva is receiving increased attention—our last meeting having been favored with a paper on this subject which has been highly commended

(and which I regret that illness prevented my attending to hear). We are no longer contented with knowing the well-worn fact that ptyalin—induces metamorphosis of amyloid bodies—that carbohydrates (heat-foods) are digested in the mouth, and nitrogenous substances (tissue-foods) in the stomach. Noting the rule that the herbivora masticate thoroughly, while carnivora bolt their food without mastication, we note also exceptions, and inquire their causes. Admitting that the presence of unaltered starch impedes gastric function, we ask to what extent? Admitting that the pancreas (so far as its glucogenic function is concerned) does not supersede, but only supplements the salivary glands, we yet want to know much concerning the qualitative differences between the salivary and the pancreatic digestions, and we want an approximation to their relative physiological worth. In a word, we want a fuller estimate of the digestion-value of the two phases of mastication—trituration and insalivation—that we may realize more justly both the uses of the teeth and the limit of their usefulness; our motive, of course, being practical—the desire to know precisely where and when conservation or destruction will best serve our patients' interests, and so become our duty.

And the flouring-mills which triturate our cereals, together with the cooks who not only macerate our food to softness, but also partially digest it—what changes have they wrought in the past, and will they work in the future, on our digestive organs and on our teeth? The relation of structure to function has occupied many pages in our journals during the past year; and in spite of certain protests which have been entered against its consideration, it appears to me a very pertinent and important subject. In the interest of operative dentistry, we want to know not only the prospects of the human teeth, but also the reasons of these prospects. We see the difference between the prognathous savage and ourselves, not only in jaw-development, but also in tooth-development, saying nothing of difference in density and hardness, of the difficulty of preserving the civilized denture as a whole, and the six-year molar in particular. We find that the number of our teeth is beginning to differ from that of the savage

—that the lateral incisor is frequently wanting, that fourth molars are unknown among us, and even third molars sometimes fail to appear. And while we want to know whether this peculiarity is a necessary result of our civilization, that we may guide our practice accordingly, we want also to know to what particular factor in our civilization it is to be attributed. True, in relation to our actual operations, the cause is less important than the fact. Whether the cause be dependence of structure on function, or whether it be diversion of nutriment from muscle and bone to nerve and brain; so long as the fact remains, and remains unalterable, our operative treatment will be nearly the same. But according as the decline of the dental organs proceeds from one cause or from another, so will hygienic prophylaxis take this or that radically different course. For purposes of operative treatment, we want mainly to know whether the present child has more teeth than he can support, whether Nature is gradually reducing the human denture to a level with its requirements, whether we should not seek to preserve a smaller number of teeth in better condition; on the other hand, for purposes of prophylactic advice, we want to know *why* human teeth are declining in size and in strength—whether it is inevitable, or whether it may be avoided by a rational system of hygienic tooth-building.

As almost every gentleman present is acquainted with me and with my practice, I shall not, I am sure, be considered as advocating a system of extraction. Conservative I have always been, and shall continue to be, but it is in place here to mention the subjects whose discussion bids fair to break down the extreme conservatism of the past, and to give a broader scope to the field of operative dentistry—to so extend it, in fact, as to bring within it a consideration of the entire life-interests of the patient.

Not that these life-interests have been unconsidered in the past. The man who has laboriously built gold crowns on all the first molars of nine-year-old children, has done so from consideration of the life-interests of his patients. But we are reaching a point whence we may contemplate those interests more fully, and may serve them more rationally. The extreme conservatist of the past *felt* that he was laboring for the patient's benefit; the moderate conservatist of the future will *see* that he does so: —and there is a wide difference between obscurely *feeling* that we do our duty, and clearly *seeing* that we do it.

Not, of course, that *all* the life-interests of our patients come under our care. Here, as before, our field shades imperceptibly into that of others. We are not to be school-masters or politicians, any more than we are to be general practitioners of medicine; but we are not to ignore the classes of interests served by the school-master and the politician, any more than we are to ignore the class served by the general medical practitioner. At the present time we consider, and in the future we will consider more broadly and more truly, the full life-interests of the patient as subserved by the healthy denture; and conversely, the effects (individual and hereditary) of the influence whereby the patient is surrounded.

Brief as has been this review of the field of operative dentistry, it has aimed to present correct views of that field from the two important stand-points. It has considered the dentist in his relation to dental disease; and has considered dentistry as offering its contribution to human welfare.

That there is just now a tendency to review limits is plain, and I believe that I have mapped the field in accordance with common sense, as well as in general harmony with the present views of the profession. The questions involved are evidently not questions of fact, but of relation; and it seems to me peculiarly important, at this time, that we should appreciate our professional relations, in order that we may clearly see what are the subjects of investigation which most demand our attention—the subjects whose consideration will in the greatest degree contribute to fit us for the full performance of the duties which our professional relations involve.

For another reason also, it is important that we should clearly see the scope of our field of usefulness, namely, that we may impress the truth in this regard upon our patients. So long as they merely come to us (as most of them do at present) that we may repair damage resulting from neglect and ignorance, they lose the better half of dentistry. They obtain simply the " pound of cure," which is notoriously of less value than the "ounce of prevention," and this loss to them of the more valuable part of our services, is due to the fact that most of them *think* what our medical friends have *said* —that we are a set of "mere mechanics." Already, however, our more intelligent patients are beginning to see the truth concerning us and our field; they come to us early for advice and care; and prophylactic dentistry has made and is making great advances, and, as opinions spread rapidly now-a-days, we may live to see the time when the office-bell will not invaribly herald the approach of a denture wrecked by ignorance and carelessness—when its sound will not recall, by universal association, the caustic saying of my native land about generous food when the horse is dead—*caballo muerto, cebada al rabo.*

